# Interleukin-1β Protects Neurons against Oxidant-Induced Injury via the Promotion of Astrocyte Glutathione Production

**DOI:** 10.3390/antiox7080100

**Published:** 2018-07-25

**Authors:** Twinkle Chowdhury, Matthew F. Allen, Trista L. Thorn, Yan He, Sandra J. Hewett

**Affiliations:** Department of Biology, Program in Neuroscience, Syracuse University, Syracuse, NY 13210, USA; tschowdh@syr.edu (T.C.); mfallen@syr.edu (M.F.A.); tlthorn@syr.edu (T.L.T.); yhe19@syr.edu (Y.H.)

**Keywords:** Interleukin 1β, neuroinflammation, neuroprotection, glutathione, astrocyte, neuronal injury, antioxidant, non-cell-autonomous protection

## Abstract

Interleukin-1β (IL-1β), a key cytokine that drives neuroinflammation in the Central Nervous System (CNS), is enhanced in many neurological diseases/disorders. Although IL-1β contributes to and/or sustains pathophysiological processes in the CNS, we recently demonstrated that IL-1β can protect cortical astrocytes from oxidant injury in a glutathione (GSH)-dependent manner. To test whether IL-1β could similarly protect neurons against oxidant stress, near pure neuronal cultures or mixed cortical cell cultures containing neurons and astrocytes were exposed to the organic peroxide, tert-butyl hydroperoxide (t-BOOH), following treatment with IL-1β or its vehicle. Neurons and astrocytes in mixed cultures, but not pure neurons, were significantly protected from the toxicity of t-BOOH following treatment with IL-1β in association with enhanced GSH production/release. IL-1β failed to increase the GSH levels or to provide protection against t-BOOH toxicity in chimeric mixed cultures consisting of IL-1R1^+/+^ neurons plated on top of IL-1R1^−/−^ astrocytes. The attenuation of GSH release via block of multidrug resistance-associated protein 1 (MRP1) transport also abrogated the protective effect of IL-1β. These protective effects were not strictly an in vitro phenomenon as we found an increased striatal vulnerability to 3-nitropropionic acid-mediated oxidative stress in IL-1R1 null mice. Overall, our data indicate that IL-1β protects neurons against oxidant injury and that this likely occurs in a non-cell-autonomous manner that relies on an increase in astrocyte GSH production and release.

## 1. Introduction

Interleukin 1β and its signaling receptor IL-1R1 are expressed at low levels in the healthy brain, where they participate in normal brain function, including learning and memory. The inhibition of IL-1β signaling prevents the induction and/or maintenance of hippocampal long-term potentiation (LTP) [1,2,3] and mice null for IL-1R1 perform poorly in the Morris water maze, a hippocampus-dependent task [3]. Additionally, there is a direct indication of the role of IL-1 signaling in the regulation of sleep and sleep patterning in both rodents and humans [4,5,6]. An equally impressive amount of literature supports its role in Central Nervous System (CNS) pathology, although a clear cause-and-effect relationship between the presence of this neuroinflammatory factor and CNS damage does not always exist (for review see: [7]). In addition, it is becoming increasingly clear that IL-1β can protect and repair CNS tissue (for review see Reference [7]). Thus, the Janus face of IL-1β may depend heavily on the context, that is, its concentration and timing, its cellular target(s), and the presence or absence of negative feedback regulators. Work from our laboratory supports this assertion. We reported previously that IL-1β enhances the expression and functional activity of the cystine/glutamate transporter (system x_c_^−^) in astrocytes, producing excitotoxic neuronal cell death in the context of energy deprivation [8,9,10,11]. However, we also find that IL-1β protects astrocytes from oxidant insults in a cell-autonomous fashion by a glutathione (GSH)-dependent mechanism [12]. Taking both in vivo and in vitro approaches herein, we investigated whether IL-1β could similarly protect neurons. We report that the loss of IL-1R1 signaling in vivo increases striatal vulnerability to 3-nitropropionic acid-mediated oxidative stress. In vitro modeling indicates that IL-1β does not protect neurons from oxidant injury directly but rather does so via stimulating the production and release of GSH from primary astrocytes.

## 2. Materials and Methods

### 2.1. Animals

This study was conducted in accordance with the National Institute of Health guidelines for the use of experimental animals and has been approved by the Institutional Animal Care and Use Committee of Syracuse University, 14-007 (approval dates: 12 September 2014–12 September 2017). CD-1 mice were obtained from Charles River Laboratories (Wilmington, MA, USA). The *il1r1* null mutant animals (Strain: B6.129S7-IL1r1^tm1lmx^/J; stock # 003245) [13] were purchased from Jackson Laboratories (Bar Harbor, ME, USA). For in vitro experiments, *il1r1* null animals were bred in parallel with animals from the background strain (stock # 000664) whose offspring were used as wild-type controls. Experimental animals for the in vivo experiments were produced from F1 heterozygous (^+/−^) breeding pairs derived from wild-type (^+/+^) female and *il1r1* null (^−/−^) male matings. Resulting ^+/+^ and ^−/−^ littermates from the F2 and F3 generations were used. The mice were provided food and water ad libitum. Up to five animals per cage were housed in a controlled temperature environment operating on a standard 12 h light/dark cycle. Investigator was blind to mouse’s genotype at the time of experimentation.

### 2.2. Cell Culture 

All cultures were kept at 37 °C in humidified 21% O_2_; 6% CO_2_-containing incubators.

Cell culture media compositions were as follows: Media stock (MS): l-glutamine-free modified Eagle’s medium (Earl’s salt; Cell Gro MediaTech, Inc., Manassas, VA, USA) supplemented with l-glutamine, glucose, and sodium bicarbonate to a final concentration of 2.0, 25.7, and 28.2 mM, respectively. Astrocyte plating medium: MS containing 10% fetal bovine serum (FBS; Hyclone Laboratories, Marlboro, MA, USA) and 10% calf serum (CS; Hyclone), 50 IU penicillin, and 50 µg/mL streptomycin (Gibco, Burlington, ON, Canada). Neuron plating medium: MS containing 5% bovine growth serum (BGS; Hyclone) and 5% CS, 50 IU penicillin, and 50 µg/mL streptomycin. Mixed culture maintenance medium: MS containing 10% CS and 50 IU penicillin/50 µg/mL streptomycin. Neuronal maintenance medium: Neurobasal medium (NB) containing 1×B27 supplement (Invitrogen, Carlsbad, CA, USA), 2 mM l-glutamine, 50 IU penicillin, and 50 µg/mL streptomycin. 

Murine: mixed cortical cell cultures, containing neurons and astrocytes, were prepared using a two-step plating method, whereby astrocytes derived from postnatal 1–3 day pups were cultured first and grown to confluency and then purified to remove microglia [14]. Dissociated neurons from embryonic day-15 mouse fetuses (0.75–1.8 million cells/mL) were then plated on top, as described in detail previously [15]. At 5 and 9 days in vitro, 250 µL of plating medium was exchanged with a mixed culture maintenance medium. To prevent microglia cell growth and induce their death, the cultures were treated with 8 µM β-d-cytosine arabinofuranoside (Ara-C) once on day 6 or 7 in vitro, which halts cell division and induces mitotic catastrophe. Neurons and astrocytes are unaffected by this treatment as the former are post-mitotic and the latter are contact-inhibited. Two days prior to experimentation, mixed cortical cell cultures were washed into media stock (2×, 250 µL exchange). Experiments were performed at 14 days in vitro. 

Near-pure neuronal cultures were derived from the cortices of embryonic day 15 CD-1 mice, as described in Reference [8]. Dissociated cortical cells were plated at a density of 1–1.4 million cells/mL on polyethyleneimine-coated plates in neuronal plating medium. Four hours later, the cell medium was exchanged fully with the neuronal maintenance medium. After two days, the neurons were treated with 1 µM of Ara-C to prevent glial cell growth. The medium was partially replenished at 4 days in vitro by exchanging 250 µL with neuronal maintenance medium made using 1×B27 supplement devoid of antioxidants (B27 minus antioxidants; Invitrogen, Carlsbad, CA, USA). Pure neuronal cultures were used at 6–7 days in vitro. 

### 2.3. IL-1β Treatment

Cells were treated with recombinant murine IL-1β (R&D Systems, Minneapolis, MN, USA) for either 24 or 48 h in an incubation buffer of MS for mixed cultures and NB for neurons both supplemented with the vehicle, 0.1% fatty-acid-free bovine serum albumin (BSA) (Sigma, St. Louis, MO, USA). As both neurons and astrocytes have functional IL-1R1 receptors, higher concentrations for the mixed cultures were utilized to ensure optimal signaling (Birgit Fogal and Sandra J Hewett, unpublished observations). 

### 2.4. Measurement of GSH

Total glutathione (GSH + GSSG) concentrations were determined using the GSH-Glo glutathione assay (Promega, Madison, WI, USA) per the manufacturer’s instruction. The media was collected for analysis of the extracellular GSH ([GSH]_e_) and GSH-Glo reaction buffer was directly added to the cells for analysis of the intracellular GSH ([GSH]_i_). The samples were diluted using the reagent to keep the relative light units within the dynamic range of the standards. To determine the total GSH levels, oxidized glutathione disulfide (GSSG) within the samples was converted to GSH with the reducing agent Tris(2-carboxyethyl)phosphine hydrochloride TCEP-HCl (final concentration = 1 mM; 10 min; 25 °C; Thermo Scientific; Waltham, MA, USA). Luciferase activity was quantified using a Synergy2 microplate reader (BioTek, Winooski, VT, USA). The GSH levels were normalized to standards prepared in the GSH-Glo reagent or MS containing l-glutamine and 0.1% fatty-acid-free BSA, respectively. The intracellular GSH was normalized to cellular protein quantified using a BCA Assay Kit (Thermo Scientific, Waltham, MA, USA) as per the manufacturer’s instructions. 

### 2.5. Drug Exposure

Tert-butyl Hydroperoxide (t-BOOH) Treatment: t-BOOH is a direct-acting organic peroxide used to induce oxidative stress; its clearance requires GSH [12,16]. A stock solution of t-BOOH (1.5 M in H_2_O; Acros Organics) was made and stored at 4 °C. t-BOOH was added to the cultures (final concentration: 1–4 mM) in the treatment media (MS for mixed cultures and NB for neurons). Experiments were terminated 0.5 to 3.5 h later and the tissue culture supernatant was removed for the measurement of lactate dehydrogenase (LDH) activity to quantify cell death (see below). Next, 3-(4,5-dimethylthiazol-2-yl)-2,5-diphenyltetrazolium bromide (MTT) was added and the extent of its reduction to formazan was used to quantify the cell survival (see below). 

MK-571: MK-571 is a potent inhibitor of MRP1, a multi-drug resistant protein transporter used by astrocytes to export GSH [12,17]. A stock solution of MK-571 (25 mM in H_2_O; Enzo Life Sciences) was made and stored at −20 °C. MK-571 was added to the mixed cultures (final concentration: 50–70 µM) in MS. After 1.5–2 h of incubation, IL-1β (final concentration = 10 ng/mL) was spiked in. Twenty-four hours later, t-BOOH was added as described in each figure’s legend. The experiments were terminated 2.5 to 3.5 h later by addition of MTT to quantify cell survival.

3-Nitroproprionic Acid (3-NP) Dosing Protocol: 3-NP is a plant toxin that produces selective striatal lesions in both experimental animals and in man via mechanisms involving oxidative stress [18,19]. 3-NP (Sigma Aldrich, St. Louis, MO, USA) was dissolved in 0.9% saline to a concentration of 25 mg/mL, adjusted to pH 7.4 with 5 M NaOH, and filter sterilized (0.2 µm Nalgene). A 3-NP stock was kept at 4 °C for no more than seven days. After five days of acclimatization handling, male mice (15–18 wks) were administered 3-NP via intraperitoneal injection twice daily with an interval of 8–12 h as follows: 20 mg/kg for two days, 30 mg/kg for three days, 40 mg/kg for three days, 50 mg/kg for three days, 60 mg/kg for one day for a total cumulative dose of 920 mg/kg. Animals completing the protocol were sacrificed by cervical dislocation under isoflurane anesthesia. The brains were snap frozen in an O.C.T. compound (Sakura Finetek USA, Torrance, CA, USA). Three separate experiments were performed over two months.

### 2.6. Measurement of Cell Death and Viability

In vitro cell death was quantitatively determined by the spectrophotometric measurement of lactate dehydrogenase (LDH) activity, as described previously [20]. Data are expressed as a percentage of total LDH activity (defined as 100%), determined by assaying the supernatant of parallel cultures exposed to 1.5 mM t-BOOH for 20–24 h. In some mixed culture experiments, cell death was additionally assessed via propidium iodide (PI; Molecular Probes, Eugene, OR, USA) staining [20]. PI (10 µg/mL) was added to culture wells for 10 min, after which it was removed by gentle washing with PBS (3 × 750 µL). Fluorescent photos were acquired by a DP73 digital color camera (Digital Video Camera Co., Olympus, Shinjuku, Tokyo, Japan) mounted on an Olympus IX50 inverted microscope outfitted with epifluorescence controlled by CellSens Standard (Olympus, Tokyo, Japan) software. Brightness and contrast were standardized for each picture. 

In vitro cell viability was quantified via colorimetric analysis of MTT (Sigma) reduction as previously described [12,21]. The percent of viable cells was quantified by normalization of experimental MTT absorbance values to values obtained from untreated control cells (i.e., highest absorbance = 100%)).

In vivo cell death was analyzed histologically. Frozen brain sections (40 µm) were collected serially from the rostrocaudal extent of each brain (+1.54 to −0.18 relative to bregma) and stained with 0.5% thionin by submersion in multiple solutions (70% EtOH, 50% EtOH, ddH_2_O, thionin, ddH_2_O, ddH_2_O, 70% EtOH, 95% EtOH, 100% EtOH, 100% EtOH, 100% Xylene, 100% Xylene), as previously described [22]. Images were captured by scanning (Epson Perfection 3170, Epson, Long Beach, CA USA) at 2400 dpi. The lesion area, identified by the absence of thionin staining, was quantified using NIH Image J at seven levels from bregma (+1.22, +1.02, +0.72, +0.52, +0.22, +0.02, and −0.18) by three individuals blind to genotype and experimental identification. For each level, the percent striatal damage (D) was calculated as a percentage of the total striatum area (T) as (D/Tx100). Area measurements were converted to volume using Cavalieri’s principle (volume = (s_1_d_1_) + (s_2_d_2_) + (s_3_d_3_) + (s_4_d_4_) + (s_5_d_5_) + (s_6_d_6_) + (s_7_d_7_)), where s = lesion surface area and d = the distance between two sections, as published in Reference [23]. The data are expressed as the percent lesion area of the striatum at all seven levels and the mean lesion volume + Standard Error of the Mean (SEM) of all seven levels derived from the mean calculated from all three individuals.

### 2.7. Statistical Analysis 

Statistical analyses were performed using GraphPad Prism Version 6.03 or 7.03 (La Jolla, CA, USA) as described in each figure legend. Percentage data were transformed by the arcsin square root function Y = arcsin[sqrt(Y/100)], before analysis because it is, by nature, non-normally distributed. In all experiments, the data are expressed as the mean + SEM. Significance was assessed at *p* < 0.05.

## 3. Results

**Figure 1:**
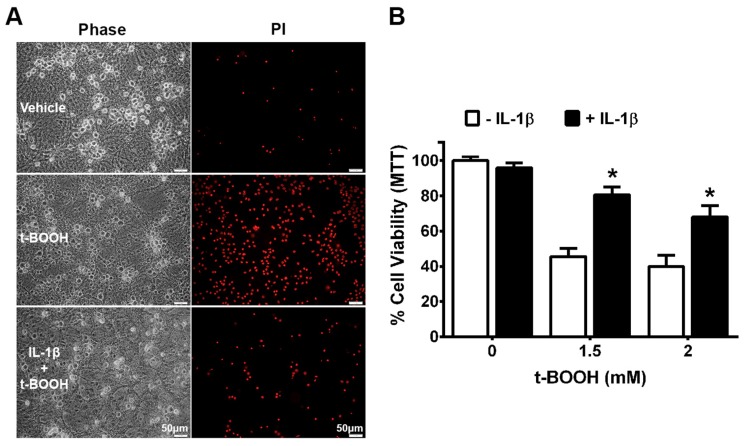
Interleukin-1β (IL-1β) pre-treatment protects mixed cultures against tert-butyl hydroperoxide t-BOOH toxicity. (**A**) Mixed cortical cell cultures were incubated with IL-1β (10 ng/mL) or its vehicle for 48 h after which they were treated with 1.5 mM t-BOOH for 2 h followed by the addition of propidium iodide (PI, 10 µg/mL) to assess for cell death. Phase contrast micrographs (left panel) and PI staining (right panel) of representative wells treated with vehicle, vehicle + t-BOOH, or IL-1β followed by t-BOOH; (**B**) Mixed cortical cell cultures were incubated with IL-1β (10 ng/mL) or its vehicle for 48 h after which they were treated with for 2.75–3.25 h followed by the addition of MTT to assess for cell survival. Data are expressed as % mean cell survival + SEM normalized to MTT values of non-treated control cultures (=100%). An asterisk (*) denotes a significant between-group difference (+ IL-1β vs. −IL-1β at each t-BOOH concentration) as determined via two-way analysis of variance (ANOVA) followed by Bonferroni’s test for multiple comparisons (*n* = 7–16 from three separate dissections; *p* < 0.001).

**Figure 2:**
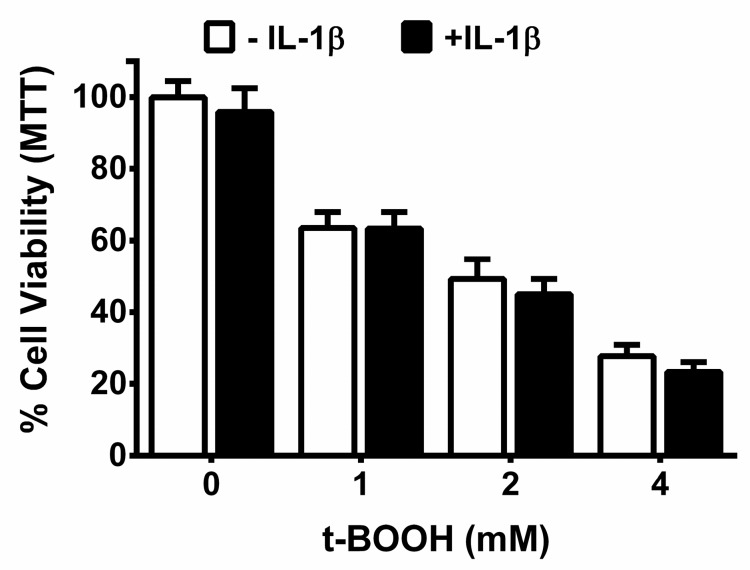
IL-1β pre-treatment does not protect pure neurons against t-BOOH-toxicity. Cortical neurons cultured in isolation were washed twice with the treatment media and incubated with IL-1β (5 ng/mL) or its vehicle for 48 h. Next, the cells were treated with t-BOOH at the indicated concentrations for 1.25–4.5 h. Cell viability was determined using the MTT assay. Data are expressed as % neuronal cell viability (mean + SEM) normalized to MTT values of the untreated group (=100%). No significant between-group differences (+IL-1β vs. −IL-1β at each t-BOOH concentration) were observed as assessed by two-way ANOVA followed by Bonferroni’s test for multiple comparisons (*n* = 6–9 from two separate dissections).

Previously, we determined that the IL-1β treatment of primary astrocytes enhanced their production and release of GSH, rendering them resistant to death induced by exposure to the organic peroxide, tert-butyl hydroperoxide (t-BOOH) [12]. Herein, we find that neurons and astrocytes in a mixed culture (Figure 1A,B)—but not neurons in isolation (Figure 2)—are also protected against t-BOOH following pretreatment with IL-1β 48 h prior to exposure. To ensure that the lack of protection in neuronal cultures was not a result of sub-optimal IL-1β concentrations, increasing concentrations of IL-1β (5–20 ng/mL; 48 h) were tested. None protected the neurons against exposure to 2 mM t-BOOH (Supplemental Figure S1). 

**Figure 3:**
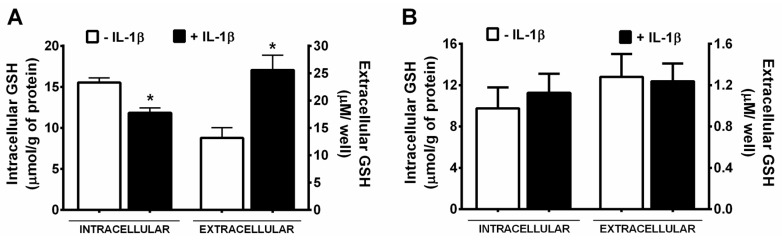
Mixed cultures, but not pure neurons, produce and release glutathione (GSH) upon stimulation with IL-1β. (**A**) Mixed cortical cultures were treated with media containing vehicle (−IL-1β; white bars) or 10 ng/mL IL-1β (+ IL-1β; black bars) (*n* = 22–24 from three to five separate dissections; well volume = 400 µL); (**B**) Neurons were treated with its vehicle or 5 ng/mL IL-1β (*n* = 9 from three separate dissections; 0.36 ± 0.024 mg protein/well). Forty-eight hours later, the total intracellular or supernatant GSH levels were measured as described in the methods. Data are expressed as mean + SEM. An asterisk (*) denotes a significant between-group difference (−IL-1β vs. +IL-1β) as determined by two-way ANOVA followed by Bonferroni’s test for multiple comparisons.

In the mixed cultures, the protective effect of IL-1β was associated with a significant increase and decrease in the supernatant and cellular GSH levels, respectively (Figure 3A). IL-1β produced no alterations in either intracellular or extracellular GSH concentrations from pure neurons (Figure 3B). 

**Figure 4:**
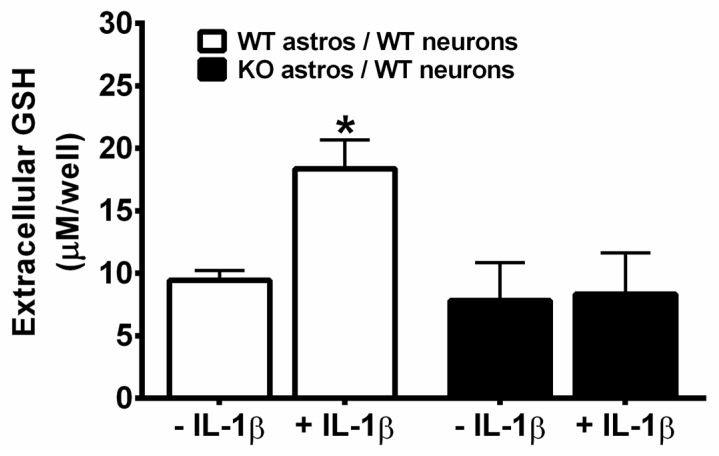
IL-1β-mediated increase in extracellular GSH in mixed cultures is dependent on astrocyte activation. Wild-type neurons plated on astrocytes cultured from *il1r1*^+/+^ or *il1r1*^−/−^ mice were treated with vehicle (−IL-1β) or 10 ng/mL IL-1β (+ IL-1β) for 48 h after which the total supernatant GSH levels were measured. The data are expressed as the mean μM GSH/well + SEM. An asterisk (*) denotes significant between-group difference (+ and −IL-1β within the same combination of chimeric culture) as assessed by two-way ANOVA followed by Bonferroni’s test for multiple comparisons (*n* = 8 each from two separate dissections; *p* = 0.001). WT: *il1r1*^+/+^; KO: *il1r1*^−/−^.

**Figure 5:**
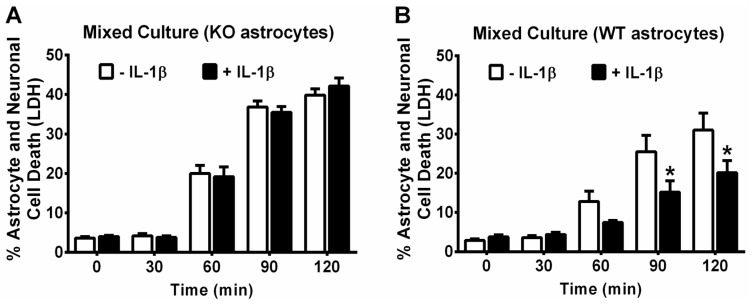
IL-1β-mediated protection against t-BOOH-toxicity in mixed cultures is dependent on astrocyte activation. Wild-type neurons plated on astrocytes cultured from (**A**) *il1r1*^−/−^ or (**B**) *il1r1*^+/+^ mice were treated with its vehicle (−IL-1β) or 10 ng/mL IL-1β (+IL-1β) for 48 h followed by treatment with 1.5 mM t-BOOH. Supernatants were collected for measurement of lactate dehydrogenase LDH at each time point, as indicated. Cultures were then washed free of t-BOOH, and medium collected again at 165 min (total time of exposure plus post-wash incubation) from each well. Values were summed and normalized to LDH released from cultures treated with 1.5 mM t-BOOH for 20–24 h (=100% cell death). Data are mean % cell death + SEM (**A**) There were no statistically significant group effects as determined by two-way ANOVA (*n* = 16 from 4 separate dissections); (**B**) *p* values were equal to 0.0035 for the IL-1β treatment effect, <0.0001 for the effect of time of t-BOOH exposure, and 0.0149 for the treatment × time interaction. An asterisk (*) depicts significant between-group difference (*p* < 0.05). Two-way ANOVA followed by Bonferroni’s test for multiple comparisons (*n* = 16 from 4 separate dissections). WT: *il1r1*^+/+^; KO: *il1r1*^−/−^.

**Figure 6:**
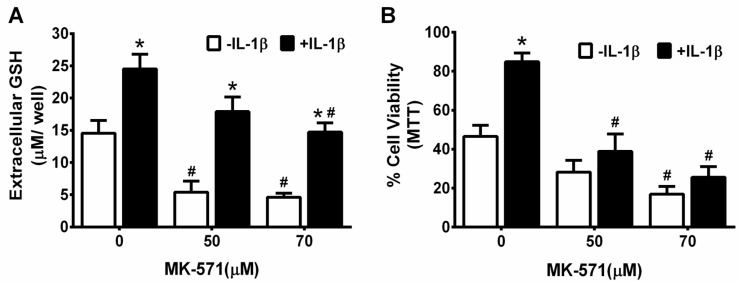
Attenuation of astrocytic GSH release abrogates the protective effect of IL-1β against t-BOOH-induced toxicity. Mixed cortical cell cultures (*n* = 8 from three separate dissections) were treated with MK-571 at the indicated concentrations for 2 h, after which IL-1β (10 ng/mL) or its vehicle was spiked in for an additional 24 h. (**A**) The supernatant was removed for measurement of the total GSH expressed as the mean μM/well + SEM, as described in the methods; (**B**) The same cells were treated with 1.5 mM t-BOOH (2.45 h) and the experiment was terminated by the addition of MTT. The results are expressed as mean % cell survival normalized to the MTT values of the non-treated control cultures (not shown = 100%). An asterisk (*) denotes significant between-group difference (−IL-1β vs +IL-1β) and a pound (#) denotes significant within-group difference (MK-571 treated with or without IL-1β) as assessed by two-way ANOVA followed by Bonferroni’s test for multiple comparisons. Significance was set at *p* < 0.05.

This IL-1β-mediated increase in extracellular GSH levels in mixed cultures was absent in chimeric mixed cultures grown with *il1r1*^−/−^ astrocytes, that is, astrocytes derived from mice lacking the IL-1β signaling receptor (Figure 4). Neurons and astrocytes in chimeric cultures containing *il1r1*^−/−^ astrocytes were also no longer protected against t-BOOH toxicity (1.5 mM t-BOOH; 2 h) by prior IL-1β treatment (10 ng/mL, 48 h), whereas those containing *il1r1*^+/+^ astrocytes were still rescued (Figure 5). These results suggest that extracellular GSH produced by astrocytes is essential for the neuroprotective effects of IL-1β. To examine this idea directly, the ability of IL-1β to protect against t-BOOH exposure following the abrogation of GSH release with MK-571 was tested. The GSH release was significantly reduced by the inclusion of MK-571 (50 µM and 70 µM) in both control and IL-1β-treated cultures, as expected (Figure 6A). The MK-571 treatment also rendered mixed cultures more vulnerable to the toxicity of t-BOOH exposure (1.5 mM, 2.45 h) and significantly attenuated the ability of IL-1β to protect them from this oxidant (Figure 6B). Thus, the neuroprotective, antioxidant potential of IL-1β rests on its ability to increase GSH production and release from astrocytes in the mixed culture. 

**Figure 7:**
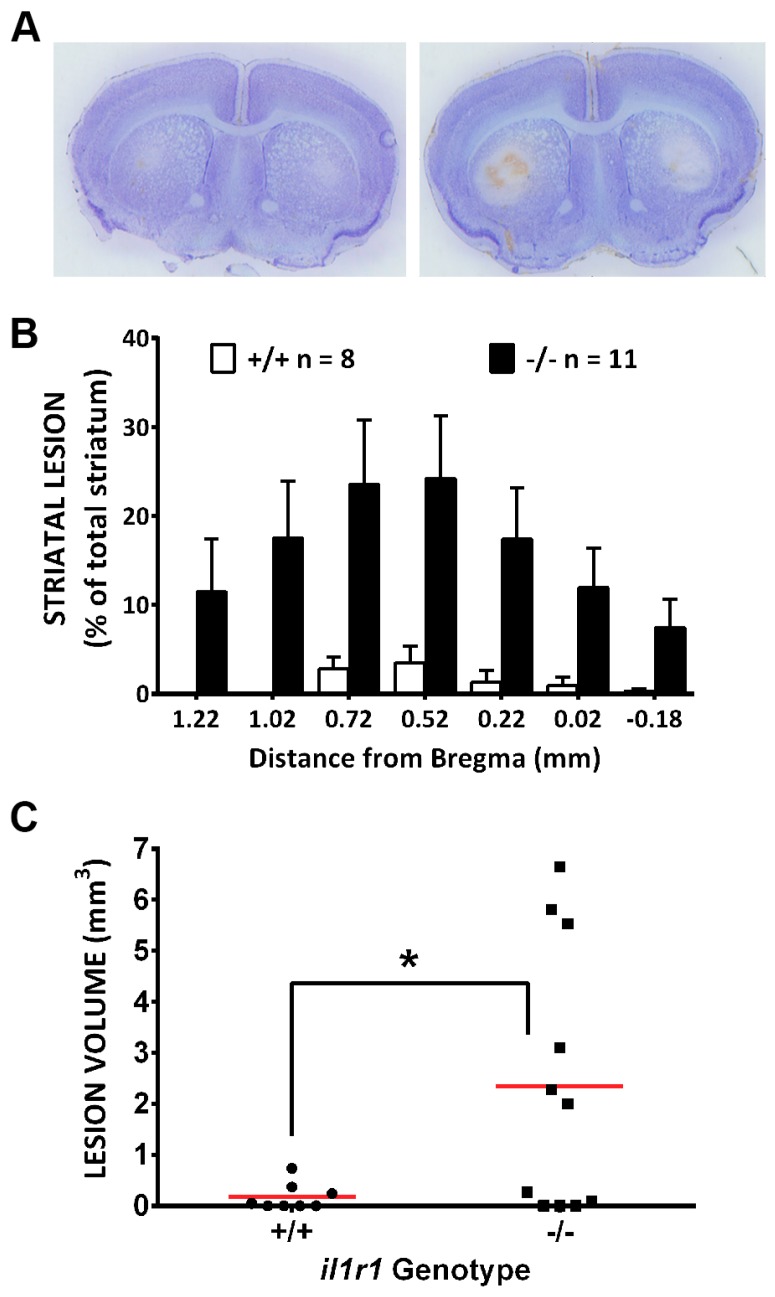
Mice deficient in IL-1R1 signaling (*il1r1*^−/−^) have larger striatal lesions following systemic 3-NP treatment as compared to their wild-type littermate (*il1r1*^+/+^) controls. Male *il1r1*^−/−^ (*n* = 11) and *il1r1*^+/+^ (*n* = 8) were injected twice daily with 3-NP as described in the methods. (**A**) Representative photomicrographs of thionin-stained sections (+0.52 from bregma) taken from 3-NP-treated *il1r1*^+/+^ (left) and *il1r1*^−/−^ mice (right); (**B**) Comparison of lesion area between *il1r1*^+/+^ and *il1r1*^−/−^ mice expressed as a percentage of total striatal area. A significant difference between genotypes occurs at every level of bregma as determined by repeated measures two-way ANOVA (*p* = 0.0163), followed by Sidaks post-hoc *t*-tests for multiple comparisons (*p* < 0.0001 at all levels); (**C**) Comparison of lesion volume between *il1r1*^+/+^ and *il1r1*^−/−^ mice determined using the Cavalieri’s method. Data points represent the lesion volume (mm^3^) for each individual mouse, while a horizontal line represents the mean mm^3^ for each group. A one-tailed *t*-test with Welch’s correction revealed a significant difference (*) between genotypes at *p* = 0.01.

Finally and importantly, these protective effects were not strictly an in vitro phenomenon as we found an increased striatal vulnerability to 3-nitropropionic acid in mice lacking the signaling receptor for IL-1β (IL-1R1 null mice) when compared to their wild-type littermate controls. Lesion size was significantly greater in IL-1R1 null mice (Figure 7). Secondly, lesion incidence—defined as the proportion of total mice from each genotype treated with 3-NP with a lesion of any size—was also greater in IL-1R1 null mice. While only 3/8 (37.5%) of *il1r1*^+/+^ mice showed histological evidence of 3-NP-induced striatal neurodegeneration, 7/11 (64%) of *il1r1*^−/−^ did (*p* = 0.13, Chi-square test). The total striatal volume was not different between the *il1r1*^+/+^ and *il1r1*^−/−^ mice (10.6 ± 0.5 and 11.8 ± 0.4 mm^3^, respectively). This increased vulnerability could not be explained by the differential metabolism of 3-NP as the striatal succinate dehydrogenase activity was inhibited to the same extent in both genotypes (data not shown). The present results indicate that loss of IL-1β signaling is detrimental to the striatum in the setting of chronic, systemic 3-NP exposure. 

## 4. Discussion

The present results demonstrate that IL-1β protects neurons against oxidant injury both in vitro and in vivo and that this likely occurs in a non-cell-autonomous manner that relies on an increase in astrocyte GSH production and release. These data fit in with a larger literature reporting the ability of IL-1β to mount protective responses in the CNS. For instance, enhanced neuronal sprouting and/or regeneration in various in vitro and in vivo model systems follow IL-1β treatment [24,25,26]. IL-1β—either endogenously produced or administered exogenously—has been shown to mediate ischemic tolerance in gerbil [27]. Treatment with IL-1β, at the same concentration as used herein in mixed cultures (10 ng/mL), protected organotypic hippocampal cultures from simulated ischemia, although hypoxic neuronal damage was enhanced [28]. IL-1β has been reported to protect against excitotoxic neuronal injury in neuronal cell cultures [29,30] and organotypic hippocampal cultures [28]. Interestingly, APP/PS1 Alzheimer’s disease mice whose astrocytes chronically overexpress IL-1β have reduced hippocampal amyloid load [31], although contextual and spatial memory impairments in control animals were evident [32,33]. Additionally, loss of IL-1β signaling exacerbated motor deficits and increased the levels of mutant huntingtin in the striatum of the N171-82Q Huntington’s disease (HD) mouse. Of note, repeated systemic administration of 3-NP—a phyto/fungal toxin that irreversibly inhibits the electron transport enzyme succinate dehydrogenase (SDH) preferentially in neurons in vivo [34]—as used herein, is often used as a chemical model of HD [18,35]. Striatal lesions subsequent to 3-NP-mediated metabolic inhibition [36,37,38] result from oxidative stress that occurs via multiple mechanisms [19,39,40]. Thus, the more numerous and larger striatal lesions in IL-1R1 null mice (Figure 7) as compared to wild-type littermate controls seen in this study, suggests that endogenous IL1-β signaling protects against 3-NP-mediated oxidative stress in vivo. 

Consistent with our in vivo results, we find that neurons and astrocytes in mixed culture treated with IL-1β also enjoy protection against oxidant injury resulting from the direct addition of the organic peroxide, t-BOOH (Figure 1). Despite the fact that both neurons and astrocytes have been shown to express IL-1R1 [41,42,43,44,45], this IL-1β-mediated protection did not extend to neurons cultured in isolation (Figure 2), indicating the need for astrocyte involvement. The importance of astrocyte signaling to the protective effects of IL-1β was directly confirmed using chimeric cultures consisting of wild-type neurons plated on astrocytes cultured from IL-1RI^−/−^ mice (Figure 5). Cell-type-specific effects of IL-1β have been reported previously. For instance, nuclear translocation of the p65 NF-κB subunit in response to IL-1β treatment of mixed hippocampal neuron-astrocyte co-cultures also occurs in astrocytes, but not neurons [46]. Interestingly, treatment of mouse primary astrocytes with IL-1β results in an NF-κB-dependent increase in the production and release of the antioxidant GSH, which subsequently rendered treated astrocytes less susceptible to oxidant injury [12]. In this study, the protective effect of IL-1β, or lack thereof, also correlated remarkably with its ability to stimulate GSH production and release from the cultures (Figure 3 and Figure 4), with again astrocytes taking center stage (Figure 4). This may not be surprising given the numerous studies demonstrating that astrocytes possess higher concentrations of GSH than neurons [47,48,49,50,51], that astrocytes protect neurons against oxidative insults [49,52,53,54,55], and that the depletion of astrocyte GSH renders neurons more susceptible to oxidative insults [49,56,57,58]. Interestingly, the release of GSH into the mixed culture medium was accompanied by a small but significant loss of intracellular GSH (Figure 3A). Since we see no change in intracellular GSH in IL-1β-treated neurons (Figure 3B), we interpret this to mean that the release from astrocytes in mixed culture is not completely matched by the synthesis, as it is when astrocytes cultures are treated with IL-1β in isolation [12]. This suggests that the dynamics of astrocyte GSH synthesis and/or release are altered by the presence of neurons. In support of this assertion, the GSH release from mixed cultures—confirmed to be astrocyte-derived in Figure 4—is 4–5 fold higher (Figure 3A) than from astrocytes cultured alone [12,59].

The cycling of GSH and/or its metabolites between astrocytes and neurons appears necessary for the maintenance of neuronal GSH levels [60,61,62]. Astrocytes reportedly facilitate neuronal GSH synthesis [47,48,63] by releasing GSH into the extracellular space [17,50,64,65] where it is cleaved to cysteinylglycine by γ-glutamyltranspeptidase [66] and hydrolyzed by a neuronal ectopeptidase to its constituent amino acids for uptake [67,68]. Our data demonstrating that the attenuation of GSH release via a block of MRP1 transport abrogates the protective effect of IL-1β in mixed culture also supports this notion of astrocyte to neuron GSH cycling. With respect to the GSH site of action, it should be noted that peroxides are certainly generated intracellularly but also in the extracellular space in vivo by infiltrating inflammatory cells [69,70,71] and/or resident microglia [72,73]; this latter process is what extracellular t-BOOH exposure to mixed cultures likely models. Additionally, hydrogen peroxide readily crosses cell membranes and this has been shown to occur through aquaporins [74], which are expressed by astrocytes and some neurons [75,76]. Finally, we previously demonstrated the increase in the intracellular reactive oxygen species in astrocytes follow extracellular t-BOOH exposure [12]. Thus, it seems likely that GSH exerts both intracellular and extracellular effects. 

How does IL-1β facilitate astrocyte production of GSH? We previously demonstrated that IL-1β enhanced system x_c_^−^ expression and thus, activity in the primary astrocytes (but not neurons [8]) in isolation [8,59] or in mixed culture [9] (also see Supplemental Figure S2). System x_c_^−^ operates as an obligate exchanger, which links the export of l-glutamate to the import of l-cystine. Rapid reduction of intracellular cystine to cysteine allows for its incorporation into proteins as well as into GSH [77,78,79]. One of the major determinates of the rate of GSH synthesis is the availability of its substrate cyst(e)ine [47,63,80]. In fact, we found that enhanced GSH export that followed IL-1β treatment is abolished in astrocytes derived from mice null for xCT, the substrate-specific light chain for the antiporter, system x_c_^−^ [59]. Unlike with xCT, we did not find a change in the steady-state mRNA levels of the regulatory enzyme of GSH synthesis, glutamate cysteine ligase (either the catalytic and modifier domain), or of MRP1 following IL-1β exposure of cultured astrocytes (Supplemental Figure S2). However, this does not rule out the possibility that IL-1β facilitates post-translational modifications leading to changes in enzymatic activity or in the trafficking of MRP1 to the plasma membrane, the latter of which has been previously shown for bilirubin-treated astrocytes [81]. If the latter is true, this could explain why MK-571 was less effective in reducing GSH efflux under IL-1β-treated conditions (Figure 6A). Moreover, various other pathways for GSH release from astrocytes have been described in the literature including gap junction hemichannels [64,65,82] and the Organic Anion-transporting Polypeptide-1, (Oatp) [83]. Whether any of these pathways are modified by IL-1β exposure was not determined. 

## 5. Conclusions

IL-1β is traditionally thought of as a classic pro-inflammatory cytokine that participates in processes leading to neurodegeneration (see References [7,84]). Contrary to this notion, we describe data demonstrating the ability of IL-1β signaling to protect neurons against oxidant injury both in vitro and in vivo. The totality of our results are consistent with the conclusion that this likely occurs in a non-cell-autonomous manner that relies on an increase in astrocyte GSH production and release. This pleiotropic nature of IL-1β in the CNS (see References [7,84]) indicates that a detailed understanding of the both the physiological, pathophysiological, and protective potential of its signaling in the brain under different contexts and conditions is necessary so that we can devise strategies to exploit its benefits while simultaneously reducing its unfavorable effects. 

## Supplementary Materials

The following are available online at http://www.mdpi.com/2076-3921/7/8/100/s1, Figure S1: IL-1β does not protect neurons cultured in isolation against t-BOOH-mediated toxicity at any concentration tested, Figure S2: Effect of IL-1β on molecules involved in GSH synthesis and export.
