# Supplementary material for: Interleukin-1β Protects Neurons against Oxidant-Induced Injury via the Promotion of Astrocyte Glutathione Production
Source: Antioxidants (Basel). 2018 Jul 25;7(8):100. doi: 10.3390/antiox7080100 (PMC6115796; doi:10.3390/antiox7080100)
Supplement: Supplementary file 1 [file antioxidants-07-00100-s001.zip › antioxidants-320854-r1-methods for figure S2.pdf]

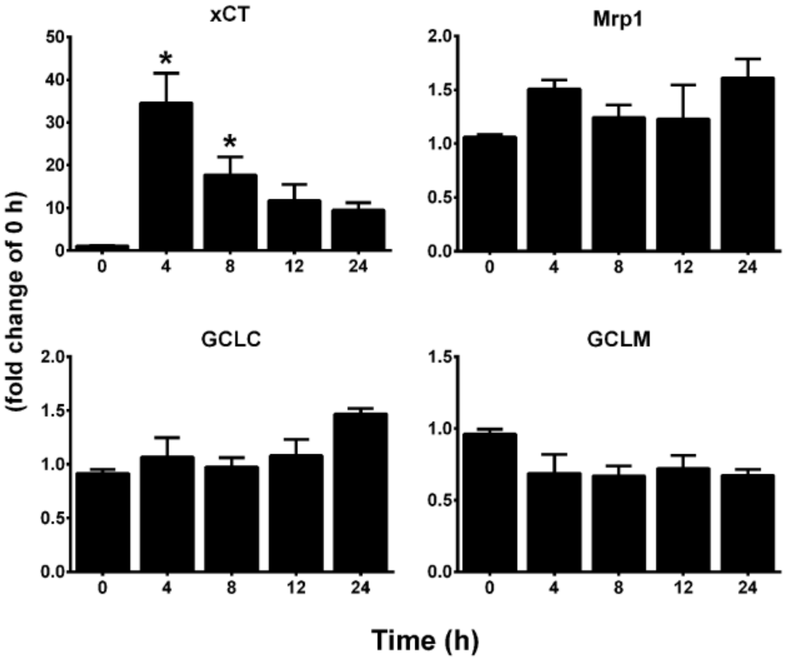

Figure S2: Effect of IL-1β on molecules involved in GSH synthesis and export. Purified astrocytes were treated with IL-1β (3 ng/mL) or vehicle (0 h) for the indicated duration and mRNA levels of the substrate specific light chain of system xc- (xCT), glutamate cysteine ligase catalytic subunit (GCLC) and modifier subunit (GCLM) as well as the GSH exporter (MRP1) were assessed via qPCR. Data are expressed as mean + SEM fold change in mRNA of genes of interest compared with untreated cells (0 h). An asterisk (*) denotes values different from 0 h as assessed by one-way ANOVA (n=3-4). Significance was set at $p < 0.05$.

Method for Figure S2: RNA was isolated and first-strand cDNA synthesized as previously described (Jackman et al., 2010). qPCR was performed using mouse-specific primer pairs [Taqman Gene Expression Assays, Applied Biosystems: systerm x_c^- light chain (xCT) (Mm00442530_m1), glutamate cysteine ligase catalytic subunit (GCLC) (Mm00802655_m1) and modifier subunit (GCLM) (Mm00514996_m1) and multidrug resistance protein 1 (MRP1/Abcc1) (Mm00456156_m1) per manufacturer's instructions. Reactions were run in the Applied Biosystems Fast Real-Time PCR System and relative quantification performed using the comparative cycle threshold method ($\Delta\Delta C_T$), where C_T values of the transcript of interest were normalized to β-actin C_T values from the same sample, then compared to a calibrator C_T value (untreated cells) to determine the relative fold increase in mRNA. β-actin C_T values were unaffected by IL-1β treatment.
